# Three-dimensional telomere profiles in papillary thyroid cancer variants: A pilot study

**DOI:** 10.17305/bjbms.2021.6639

**Published:** 2021-11-30

**Authors:** Aline Rangel-Pozzo, Tinuccia Dettori, Daniela Virginia Frau, Federica Etzi, John Gartner, Garbor Fisher, Roberta Vanni, Sabine Mai, Paola Caria

**Affiliations:** 1 Department of Cell Biology, Research Institute of Oncology and Hematology, CancerCare Manitoba, University of Manitoba, Winnipeg, Canada; 2 Department of Biomedical Sciences, University of Cagliari, Monserrato, Italy; 3 Department of Biomedical Sciences-Histology, University of Sassari, Sassari, Italy; 4 Department of Pathology, Max Rady College of Medicine Rady Faculty of Health Sciences, University of Manitoba, Winnipeg, Canada

**Keywords:** Papillary thyroid cancer, non-invasive follicular thyroid neoplasm with papillary-like nuclear features, follicular adenoma, 3D telomere profiles

## Abstract

Besides the two main histologic types of papillary thyroid carcinoma (PTC), the classical PTC (CL-PTC) and the follicular variant PTC (FV-PTC), several other variants are described. The encapsulated FV-PTC variant was recently reclassified as non-invasive follicular thyroid neoplasm with papillary-like nuclear features (NIFTP) due to its similarities to benign lesions. Specific molecular signatures, however, are still unavailable. It is well known that improper DNA repair of dysfunctional telomeres may cause telomere-related genome instability. The mechanisms involved in the damaged telomere repair processing may lead to detrimental outcomes, altering the three-dimensional (3D) nuclear telomere and genome organization in cancer cells. This pilot study aimed to evaluate whether specific 3D nuclear telomere architecture might characterize NIFTP, potentially distinguishing it from other PTC histologic variants. Our findings demonstrate that 3D telomere profiles of CL-PTC and FV-PTC were different from NIFTP and that NIFTP more closely resembles follicular thyroid adenoma (FTA). NIFTP has longer telomeres than CL-PTC and FV-PTC samples, and the telomere length of NIFTP overlaps with that of the FTA histotype. In contrast, there was no association between BRAF expression and telomere length in all tested samples. These preliminary findings reinforce the view that NIFTP is closer to non-malignant thyroid nodules and confirm that PTC features short telomeres.

## INTRODUCTION

Thyroid cancer is one of the most common malignant endocrine neoplasms and papillary thyroid carcinoma (PTC) constitutes approximately 80% of all thyroid cancer cases [1]. The cancer genome atlas study [2] has demonstrated a strong correlation between genetic alterations and histologic phenotypes of thyroid neoplasia, resulting in the identification of two distinct molecular subgroups: The BRAFV600E-like nodules, which show the true papillary architecture, and RAS-like nodules, with a follicular-pattern that includes follicular thyroid adenoma (FTA), follicular carcinoma, and follicular variant PTC (FV-PTC). Recently, the non-invasive follicular thyroid neoplasm with papillary-like nuclear features (NIFTP) variant [3], a very low-risk thyroid tumor previously known as an encapsulated non-invasive follicular variant PTC (EFVPTC), has been included in the RAS-like subgroup. However, no specific molecular signatures have been identified [4,5]. Although the vast majority of nodules classified as NIFTP have an overall clinical favorable behavior, lymph node metastasis has been reported in a minority of cases [4], pointing to the need for the identification of new accurate potential diagnostic parameters.

It is well known that telomeres have an essential role in preserving chromosome stability and integrity. Intact telomeres prevent end-to-end fusions, degradation of the chromosome ends, and contribute to the adequate chromosome positioning within the nucleus [6]. Studies using quantitative three-dimensional (3D) telomere imaging have shown differences in the 3D telomere architecture in the nucleus of cancer cells compared to the pattern observed in the normal cell nucleus [7-10]. Recently, we highlighted the importance of telomere-related genomic instability during the tumorigenesis of PTC. By selecting PTC-derived cell lines characteristic of PTC tumors in the BRAF^V600E^-like subgroup, we demonstrated that isolated cancer stem-like cells (representing an early cancer-promoting subpopulation) had a trend toward lower telomere shortening compared to the corresponding parental cells (representing the tumor bulk cells) [11]. Literature data highlight that improper DNA repair of dysfunctional telomeres can cause telomere-related genome instability [12]. As a result, harmful outcomes can occur that alter the 3D nuclear telomere and genome organization in cancer cells [9]. On these bases, considering that the biological features of NIFTP are still unclear, possibly due to the highly variable incidence (<1-28% of all PTC) and the lack of well-established differentiation criteria [13], we designed a pilot study to investigate the potential role of 3D telomere imaging and nuclear architecture in the further characterization of this rare thyroid tumor. Although with a reduced number of cases, this pilot study indicates that NIFTP has specific 3D nuclear telomere architecture and suggests that the specific 3D pattern observed may provide an additional parameter in the differential diagnosis of indolent thyroid nodules, thereby preventing unnecessary patient treatment.

## MATERIALS AND METHODS

### Patient samples

A total of 15 primary thyroid tumor tissue samples were obtained from the Health Sciences Centre (Winnipeg, Manitoba, Canada). The tumor and normal adjacent tissue (NAT) of formalin-fixed paraffin-embedded (FFPE) sections (5 mm) were circled with a pen after review of the corresponding hematoxylin- and eosin-stained sections by two pathologists (JG and GF). The experiments were blinded to the tumor characteristics and outcome data. The patient cohort included in this study was composed of 3 (20%) men and 12 (80%) women ages 31-67, with a median age of 51 years. Surgical specimens were classified according to the World Health Organization classification [14]. Clinical, pathological, and molecular characteristics of the 15 patients are shown in Table 1.

**TABLE 1:**
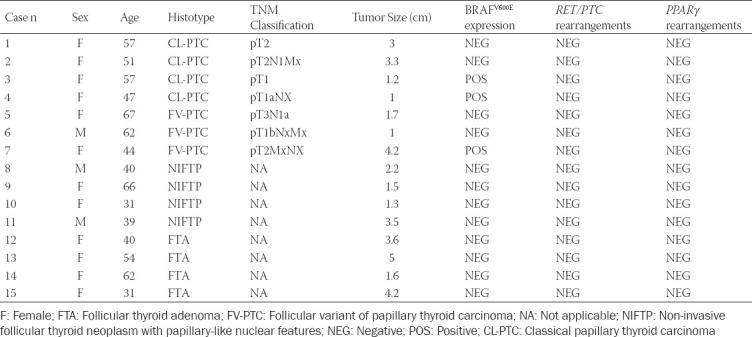
Clinical, pathological, and molecular characteristics of patients

### 3D quantitative fluorescence in situ hybridization (Q-FISH)

Nuclei from 5-mm thyroid tissue sections underwent 3D quantitative fluorescence *in situ* hybridization Q-FISH with a peptide nucleic acid-Cy3-telomere probe-(TTAGGG)n (DAKO, Glostrup, Denmark). The hybridization procedure was performed as previously described [15]. The 3D image analysis was carried out on 100 nuclei per tumor sample using AxioImager Z2 microscope (Carl Zeiss Canada Ltd). A 63x/1.4 oil objective lens (Carl Zeiss Canada Ltd.) were used for image acquisition. Eighty z-stacks were acquired at a sampling distance of x, y: 102 nm, and z: 200 nm for each slice to create optical sections. A constant acquisition time of 300 milliseconds for Cy3 (telomeres) was used, while the exposure times for the DAPI (nuclei) varied for each sample. The constrained iterative algorithm was used for deconvolution [16]. Deconvolved images were analyzed using the TeloView® v1.03 software program (Telo Genomics Corp., Toronto, ON, Canada) [17]. TeloView® measures six different telomere parameters in each cell, generating specific 3D telomere profiles for each thyroid sample examined [11,18].

### BRAF expression and RET/PTC and PPARg rearrangements

BRAFV600E protein expression was detected using the anti-BRAFV600E (clone VE1) mutation-specific mouse monoclonal antibody (Abcam ab228461 Milano, Italy) in accordance with the protocol utilized in the previous studies [19]. Sequencing-validated *BRAF^V600E^* mutation positive (seven samples) and negative (three samples) PTC cases [20] were used as positive and negative controls, respectively, to test clone VE1 antibody. Normal thyroid tissue from the tumor-free contralateral lobe of five PTCs was also used as control [21]. The Image J software (US National Institutes of Health, USA) was used to determine the fluorescence intensity as previously described [22]. All BRAF positive controls showed BRAF protein expression, with mean fluorescence intensity of 100 (100 ± 10 SD) arbitrary units (a.u) ranging from 81.5 to 113, and this range of values was considered to assign the presence of BRAF^V600E^ mutation in our samples. In BRAF negative cases, the mean fluorescence intensity was 19 a.u (19 ± 0.7 SD), ranging from 19.8 to 20.7. This range of values was used to assign the absence of BRAF^V600E^ mutation in our samples. Mean fluorescence intensity in normal thyroid tissues was in the range of BRAFV600E negative controls, corroborating this approach.

Images were obtained with an epifluorescence microscope (Olympus BX41) and charge-coupled device camera (Cohu), interfaced with the CytoVysion system (software 2.81 Applied Imaging, Pittsburg, PA, USA). In each section, tumor and normal area were evaluated, and ten randomly selected fields for each sample were acquired with a ×100 objective.

The presence of RET/PTC and PPARg rearrangements was tested by FISH, using the dual-color break-apart strategy, as previously described [21].

### Ethical statement

Informed written consents were collected from all the patients in accordance with the declaration of Helsinki. The protocol of this study was approved by the Health Research Ethics Board on human studies from the University of Manitoba, Canada, (HS21723; H2018:156).

### Statistical analysis

To compare telomere parameters among different histology, nested factorial analysis of variance was used. We also compared telomere features of cells from NAT and from tumor area for each patient with a different histology using the Wilcoxon rank sum tests. Significance levels were set at *p* = 0.05.

## RESULTS

### 3D telomere profiles in different PTC histotypes

**FIGURE 1:**
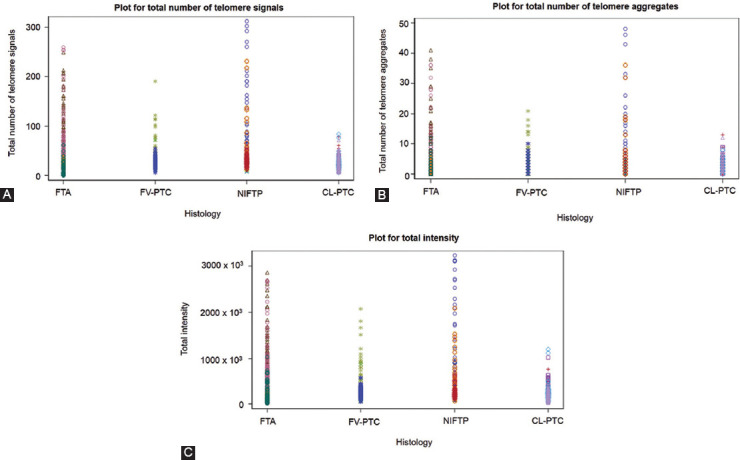
Dot plot of telomere parameters for FTA, FV-PTC, NIFTP, and CL-PTC cases. Each cell analyzed per histotype is represented in the graph. (A) Total number of telomere signals – a sum value representing the number of telomeres found in each cell population. (B) Total number of telomere aggregates – telomeres in proximity forming clusters that cannot be further resolved at an optical resolution limit of 200 nm – which, functionally, are fused telomere signals or telomeres in close illegitimate proximity able to engage in recombination events. (C) Total telomere signal intensity (proportional to telomere length). The x-axis shows values for each parameter and the y-axis refers to the cells analyzed in each histotype. FTA: Follicular adenoma; FV-PTC: Follicular variant papillary thyroid carcinoma; NIFTP: Noninvasive follicular thyroid neoplasm with papillary-like nuclear features; CL-PTC: Classical-papillary thyroid carcinoma.

**FIGURE 2:**
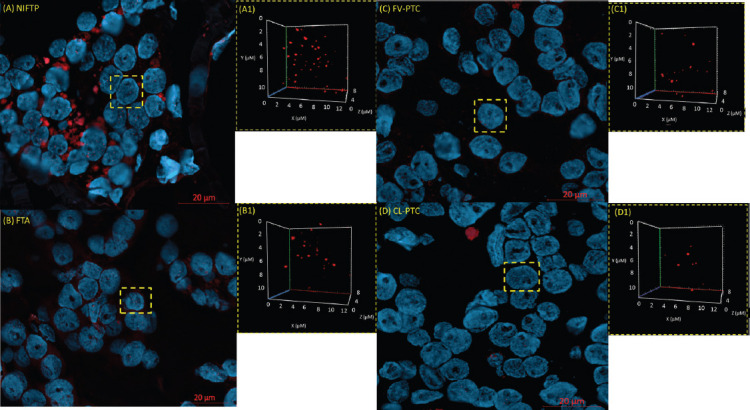
Differences in the 3D nuclear telomere architecture between NIFTP (A), FTA (B), FV-PTC (C), and CL-PTC (D). Representative nuclei, counterstained with DAPI (blue), are shown for each histotype. Cy-3 labeled telomeres appear as red signals in the 3D figures in A1, B1, C1, and D1. NIFTP: Non-invasive follicular thyroid neoplasm with papillary-like nuclear features; FTA: Follicular adenoma; FV-PTC: Follicular variant papillary thyroid carcinoma; CL-PTC: Classical-papillary thyroid carcinoma, 3D: Three-dimensional.

In the present pilot study, we examined 15 FFPE thyroid neoplasms comprising classical PTC (CL-PTC) (n = 4), FV-PTC (n = 3), NIFTP (n = 4), and FTA (n = 4). We compared their respective telomere parameters across and found a pattern differentiating FTA and NIFTP from FV-PTC and CL-PTC. FV-PTC and CL-PTC presented lower numbers of telomere signals, lower numbers of telomere aggregates, and lower total intensity when compared with FTA and NIFTP (Figures 1A-C and 2).

**FIGURE 3:**
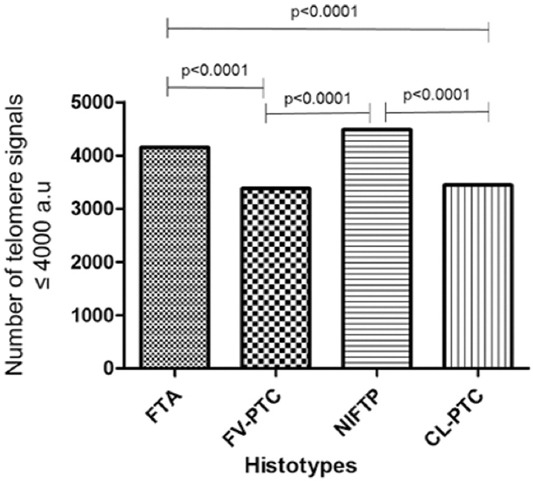
Bar plot of the differences in number of telomeres ≤ 4000 a.u (arbitrary units) between CL-PTC, FV-PTC, FTA, and NIFTP. Cells with intensity of ≤4000 a.u. represent very short telomeres. The x-axis assigns one box for each cell population analyzed (results of all analyzed cells). The y-axis refers to the number of telomeres ≤4000 a.u. (showing only the significant *p* values). CL-PTC: Classical-papillary thyroid carcinoma; FV-PTC: Follicular variant papillary thyroid carcinoma; FTA: Follicular adenoma; NIFTP: Non-invasive follicular thyroid neoplasm with papillary-like nuclear features.

TeloView® analysis, creating distributions based on the frequency of telomere signals with specific intensity, enabled us to distinguish four quartiles, in arbitrary units (a.u.), indicating four cell subpopulations: Cells with very short telomeres (≤4000 a.u.), cells with short telomeres (4001-7000 a.u), cells with medium telomeres (7001-13000 a.u), and cells with large telomeres (>13.000 a.u.). The frequency distribution of very short telomeres (≤4000 a.u) showed a significant difference between the histotypes FTA and NIFTP versus CL-PTC and FV-PTC histotypes (Figure 3). Indeed, no significant differences were observed between FTA and NIFTP or between CL-PTC and FV-PTC, NIFTP paralleling FTA telomere length while having longer telomeres compared to CL-PTC and FV-PTC. The analysis of the 3D telomere profiles in NAT showed that the most of the telomere parameters were different between NAT and FTA tumor area and NAT and NIFTP tumor area, whereas number of telomeres signals and total intensity were significantly different between NAT and CL-PTC tumor area, and the average intensity was different between NAT and FV-PTC (Table 2).

**TABLE 2:**
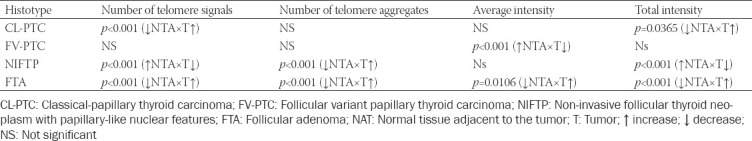
Three-dimensional telomere parameters of thyroid tumor versus normal to adjacent tumor tissue

### BRAF^V600E^ expression and RET/PTC and PPARγ rearrangements

**FIGURE 4:**
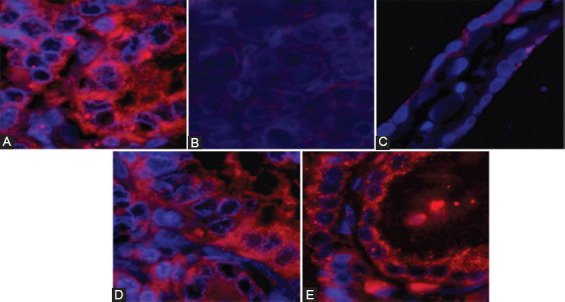
Representative images of BRAFV600E (VE1) immunofluorescence staining. BRAFV600E – mutant PTC case (A), BRAFV600E – wild type PTC (B), and normal thyroid tissue (C), CL-PTC and FV-PTC with an unequivocal diffuse cytoplasmic staining with the VE1 antibody in the majority of tumor cells (D and E). PTC: Papillary thyroid carcinoma.

BRAF protein expression was indicative of BRAFV600E mutation in three out of 15 cases. The three cases (two CL-PTC and one FV-PTC) showed unequivocal diffuse cytoplasmic antibody fluorescence intensity between 81 and 86.5 a.u. in the majority of tumor cells (Figure 4). Analysis for possible RET and PPARg rearrangements accomplished by dual-color break-apart probe FISH was negative in all cases.

## DISCUSSION

Noninvasive follicular thyroid neoplasm with papillary-like nuclear features (NIFTP), previously known as EFVPTC, displays cells with features that look like PTC but are considered to have an extremely low risk of adverse outcomes. NIFTP tumor diagnosis must meet very stringent morphological criteria since no specific markers are available nor molecular signatures have been defined, although NIFTP falls among the RAS-like papillary tumors [4]. In this context, we designed a pilot study to investigate the 3D telomere profiles of malignant PTC variants and benign FTA to assess whether NIFTP 3D telomere profiles could be evaluated as a possible ancillary parameter in the characterization of this low-risk tumor variant.

We counted lower numbers of telomeres and shorter telomeres in CL-PTC and FV-PTC variants than NIFTP and FTA. This result suggests the decrease in the number of telomeres as a consequence of telomere shortening since fewer telomeres (TTAGGG) repeats would be available for probe binding. This observation is in line with the previous studies indicating the association of short telomeres with classic and follicular variants of PTC and long telomeres with benign thyroid nodules [23,24]. Dot plot profile showing higher numbers of telomere aggregates for FTA and NIFTP compared to FV-PTC and CL-PTC cases corroborates this view. Indeed, the aggregates observed in FTA and NIFTP may represent the telomere associations seen in classical cytogenetic studies [25,26]. Our data, indicating that the investigated NIFTP has telomere signatures similar to FTA and dissimilar from CL-PTC and FV-PTC, reinforce the idea that NIFTP is more closely related to benign thyroid neoplasm rather than to malignant lesions [27,28]. In addition, our NIFTP cases were negative for BRAFV600E, according to the lack of this mutation in NIFTP [29].

Considering that telomeres length in healthy tissue can vary significantly from one patient to another, we also analyzed the 3D telomere profiles in NAT. In general, the most of the 3D telomere profile parameters differed between NAT and tumor areas of FTA and NIFTP, indicating a clear-cut separation between the profiles of normal tissue cells and cells of these two nodule variants contained within the tumor capsule. On the contrary, in the malignant tumors, among the 3D telomeres parameters analyzed, we found that only the number of telomeres and total intensity were significantly different between NAT and CL-PTC tumor areas. In contrast, between FV-PTC and the respective NAT, only the average intensity was different. The finding of this altered feature in the surrounding-tumor cells with apparently normal morphology suggests that the alteration is an early molecular event preceding changes visible at the histological level.

## CONCLUSION

In summary, our preliminary data show that 3D telomere organization of NIFTP is similar to that of benign FTA, and it is dissimilar from that of classical and follicular variant PTC. These findings are in line with the view that NIFTP is a lesion closer to non-malignant thyroid nodules and confirmed that short telomeres are a feature of PTC. It is of note that, in keeping with the stringent criteria for NIFTP diagnosis, our NIFTP samples are negative for both BRAF and RET alterations.

Our first study of 3D nuclear telomere organization in PTC primary tumor variants provides a strong rationale for additional studies in a larger cohort. If confirmed, our findings are a promising tool to be considered among the strict parameters required to reach the exact diagnosis of NIFTP, enabling predicting the outcome of this tumor and patient-specific clinical management.
